# A Physics-Informed Automatic Neural Network Generation Framework for Emerging Device Modeling

**DOI:** 10.3390/mi14061150

**Published:** 2023-05-29

**Authors:** Guangxin Guo, Hailong You, Cong Li, Zhengguang Tang, Ouwen Li

**Affiliations:** School of Microelectronics, Xidian University, Xi’an 710071, China

**Keywords:** emergingdevice modeling, neural network, physical informed, automated machine learning (AutoML), compact model, semiconductor device, circuit simulation, field-effect transistor (FET)

## Abstract

With the rapid development of semiconductor technology, traditional equation-based modeling faces challenges in accuracy and development time. To overcome these limitations, neural network (NN)-based modeling methods have been proposed. However, the NN-based compact model encounters two major issues. Firstly, it exhibits unphysical behaviors such as un-smoothness and non-monotonicity, which hinder its practical use. Secondly, finding an appropriate NN structure with high accuracy requires expertise and is time-consuming. In this paper, we propose an Automatic Physical-Informed Neural Network (AutoPINN) generation framework to solve these challenges. The framework consists of two parts: the Physics-Informed Neural Network (PINN) and the two-step Automatic Neural Network (AutoNN). The PINN is introduced to resolve unphysical issues by incorporating physical information. The AutoNN assists the PINN in automatically determining an optimal structure without human involvement. We evaluate the proposed AutoPINN framework on the gate-all-around transistor device. The results demonstrate that AutoPINN achieves an error of less than 0.05%. The generalization of our NN is promising, as validated by the test error and the loss landscape. The results demonstrate smoothness in high-order derivatives, and the monotonicity can be well-preserved. We believe that this work has the potential to accelerate the development and simulation process of emerging devices.

## 1. Introduction

With the development of semiconductor technology, fabricating and evaluating new transistors is time-consuming and expensive. Compact models serve as the bridge between device process technology and electronic integrated circuit (IC) design. It is essential to quickly complete transistor modeling accurately to save time and costs [1]. Standard compact models of transistors (e.g., BSIM-CMG [2], GSIM-IMG [3], and PSP [4]) are widely used in industry, but they have difficulty modeling new emerging devices. As transistors are scaled, more new unideal effects and quantum mechanical effects appear. These new challenges increase the difficulty of modeling new emerging devices for three reasons: (a) the traditional standard FET models cannot well-capture the electrical characteristics of emerging devices, (b) developing the physics-based model equation requires a long time and expertise, and (c) for equation-based models, it is still challenging to fully automate the model parameter extraction process while achieving a very high fitting accuracy [5]. In the previous studies [6,7,8,9,10,11,12,13,14,15,16], Neural Networks (NN) show promising accuracy in emerging device modeling. However, NN-based device modeling suffers from two main issues: unphysical behaviors and needing NN expertise [5]. The unphysical issues, such as un-smoothness, non-zero drain current ($I_{d}$) at $V_{DS}$ = 0, and lacking monotonic dependency, are blocking the adoption of NN-based methods by the industry scene. The requirements of expertise issue consumes lots of time to try out an appropriate NN with high accuracy and lightweight.

Several approaches have been proposed to solve the unphysical issues of NN-based modeling. For instance, Li et al. [6] used a two-portion NN with different activation functions for $V_{d}$ and $V_{g}$, but it can only handle the terminal voltage and not other electrical inputs such as gate length ($L_{g}$). Kao et al. [11] combined physics-based compact models (e.g., BSIM [2]) with NN output, but it relies on established compact models and is computationally complex. Wang et al. [5] used a symmetry transform function to obtain a smooth curve, but it cannot handle an unsymmetrical source/drain scene [17]. Huang et al. [18] incorporated a physical-relation-neural-network to map between device parameters and surface potential, then constructed $I_{d}$ by mathematical equations, which may induce additional errors for emerging devices. Tung et al. [10] used a loss function to smooth the output, but this approach only works on oversampled data. When the input electrical parameters are increased, the oversample may result in an unacceptable data size. Few works incorporate the monotonic dependence on the NN network, for instance, drain/source current ($I_{ds}$) increases when $V_{gs}$ increases. When the data sample is less or the variable relationship is not clear, it is necessary to set the monotonic dependency between the input and output electrical parameters of the NN.

The commonly used method to obtain an appropriate NN structure is based on the trial-and-error method. The issue of needing NN expertise has confused semiconductor background researcher a lot. Wang et al. [5] used SPICE simulation turn-around time to find an appropriate structure that balances accuracy and speed, which is less of a guidance and time-consuming process. They increased the NN size when the accuracy was low and reduced the NN size when the SPICE simulation time was high. This may lead to a loop when there is no solution. Tung et al. [10] tested the relationship between nodes number and speed using grid search. Additionally, they searched parameters using the trial-and-error method.

To summarize, the primary challenges of NN-based device modeling include the following: (1) Requiring expertise in neural networks to establish an appropriate structure. (2) Addressing unphysical issues associated with NN-based modeling, including: (a) Ensuring smooth differentiability of $I_{d}$ with respect to $V_{gs}$ and $V_{ds}$. (b) Establishing a monotonic $I_{d}$ curve. (c) Ensuring that $I_{d}$ equals 0 when $V_{d}$ equals 0. (d) Incorporating both symmetric and asymmetric drain/source scenarios. (e) Leveraging existing device modeling knowledge.

**Figure 1:**
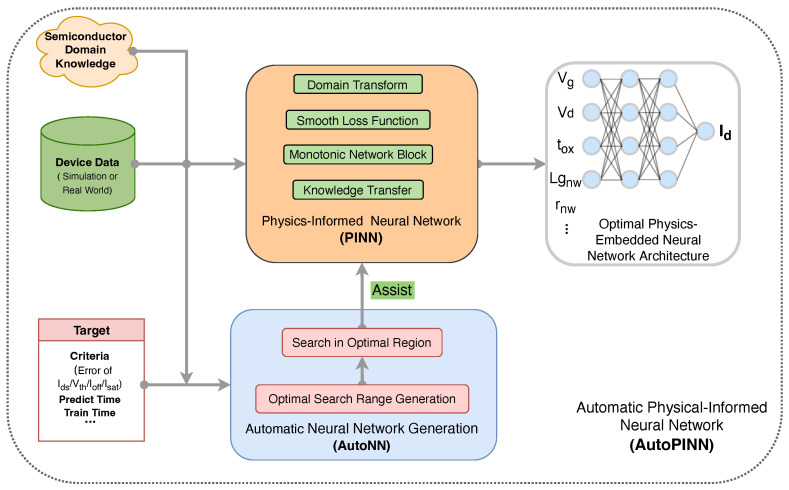
Physical-Informed automatic neural network generation framework. The PINN is embedded with physical information from device data and semiconductor knowledge. The AutoNN assists PINN to find an optimal structure to meet the target.

In this paper, an Automatic Physical-Informed Neural Network (AutoPINN) generation framework is proposed, as shown in Figure 1. This framework is composed of two parts: Automatic Neural Network (AutoNN) and Physics-Informed Neural Network (PINN). Compared with other general NN methods, the PINN method has better physical behavior because of taking the physical information of device modeling into consideration. Compared with other general AutoNN methods, the AutoNN is optimized for our PINN regarding device modeling. It can substantially decrease the search time during the NN architecture optimization, according to the complexity of input device data.

This framework takes device data, semiconductor domain knowledge (e.g., monotonic relationship between $V_{ds}$ and $I_{ds}$), and the optimization target as input. Then, it generates a device modeling neural network, with optimal structure and physical information embedded. The AutoNN assists PINN to find an optimal structure without human involvement. It can solve the expertise issue mentioned before. To overcome the unphysical issues, the PINN is introduced. The PINN embeds physical information with a few key technologies, such as Domain Transform, Smooth Loss Function, Monotonic Network Block, and Knowledge Transfer. The Domain Transform makes $I_{d}$ smooth and differentiable to $V_{ds}$ by increasing the density near the $V_{ds}$ = 0. The functions have the ability to handle both symmetry and un-symmetry drain/source scene. It also transforms the optimization target to a new one, which can ease the burden of NN fitting. The Smooth Loss Function takes not only the optimization target, but also the derivatives and other factors into consideration. It makes the total $I_{d}$ curve smooth and differentiable. The Monotonic Network Block is used to obtain the monotonic behavior by constraining the weight of NN as non-negative. The information from other devices can be transferred to new device modeling by Knowledge Transfer. It can speed up the training convergence process and obtain better physical behavior.

The contributions of this paper are summarized as follows:

(1) In this paper, a physics-informed neural network (PINN) is proposed, which can embed physical device information into neural networks (NN) to overcome non-physical behaviors and improve accuracy in compact modeling. The techniques proposed include the Domain Transform functions, Smooth Loss Function, Transfer Knowledge, and Monotonic Network Block. These techniques aim to make NN-based modeling practical.
(2) This paper proposes a two-step Automatic Neural Network (AutoNN) method for optimizing PINN structure. The framework involves two steps: (a) generating a small range of PINN parameters according to the complexity of electrical features, and (b) finding the optimal PINN structure based on accuracy and speed. The AutoNN assists PINN to improve accuracy without human involvement.
(3) Evaluated on the TCAD-simulated gate-all-around transistor (GAAFET) device, this framework can achieve an error of less than 0.05%. The framework outperforms an ensemble learning result, achieving a 72.2% reduction in the error of the drain current ($I_{d}$) compared to the ensemble method.

The rest of this article is divided into the following sections. Section 2 presents several key techniques to embed physical information into NN. In Section 3, the optimization of PINN is described using a two-step AutoNN technique to find the optimal architecture based on user-defined targets. Then, in Section 4, we present the experimental results of our framework evaluated on the GAAFET data. Finally, in Section 5, we summarize our key conclusions.

## 2. Physical-Informed Neural Network

Although the neural network (NN) has the power of universal approximation, there are still some challenges to bringing NN-based device modeling methods to practical use. The most important is the non-physical behaviors of the NN-based model. This section focuses on the techniques proposed to embed physics information into NN to overcome the barriers and improve the accuracy of device modeling. Physical behaviors play a crucial role in making the results more reasonable and practical. Moreover, it can induce accuracy improvement. The key techniques proposed, including the Domain Transform function, Monotonic Network Block, Smooth Loss Function, and Transfer knowledge, are designed to effectively integrate physical behaviors into NN-based device modeling methods.

### 2.1. Smooth Loss Function

The loss function is a critical factor in determining the accuracy of a neural network, as it guides the direction of optimization. It is also an intuitive way to incorporate physical information into the network. Therefore, it is essential to define an appropriate loss function that takes into account both accuracy and physical behavior. The proposed loss function for the PINN is defined in a smooth and accurate way in Equations (1) and (2).

(1) $$Loss=Err\left(y_{true}-y_{pred}\right)+\alpha \times Err\left(e^{y_{true}}-e^{y_{pred}}\right)+\beta \times Err\left(\frac{Diff_{I_{d}}}{Diff_{V_{g}}}\right)+\gamma \times Err\left(\frac{Diff_{I_{d}}}{Diff_{V_{d}}}\right)$$

(2) Err=RMSE

where α,β,γ are the weight that controls the importance of each component in the loss function. The first component aims to decrease the error in the logarithmic scale to accurately model the sub-threshold region. To improve the accuracy of the saturated region, the second component of Equation (1) considers the error in the original numerical scale. Additionally, the smoothness of the current-voltage (I−V) curve is an important physical behavior that can be integrated into the loss function by adding the derivative of $I_{d}$ with respect to the input voltage ($V_{g}$ and $V_{d}$) as the third and fourth components, respectively. It is important to mention that TCAD simulations or hardware measurements produce discrete numeric values, and therefore, a numerical approximation is employed to represent the derivative of $I_{d}$ with respect to $V_{g}$ and $V_{d}$.

**Figure 2:**
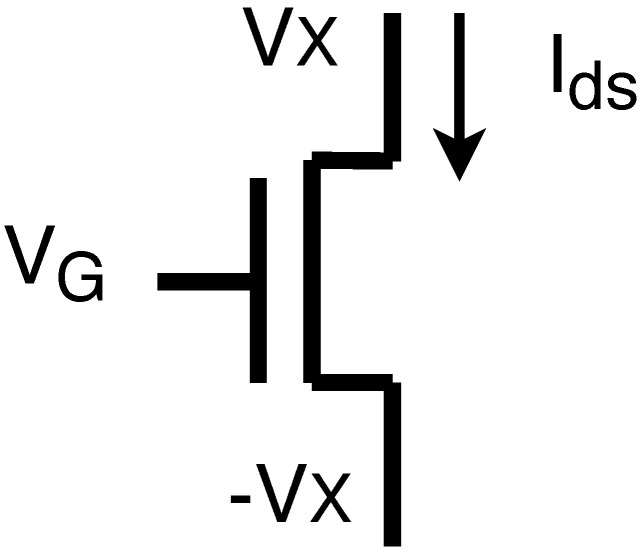
Gummel symmetry test setup circuit.

The Gummel Symmetry Test (GST) is a well-established method used to evaluate the smoothness and symmetry of the current-voltage characteristics of a device. This method was first introduced by Gummel [19], and it has since become widely adopted in the field. Figure 2 shows a circuit with a GAAFET device, the GST involves setting a specific voltage ($V_{G}$) on the gate and varying another voltage ($V_{X}$) to measure the current ($I_{d}$) flowing through the device. Then, the smoothness and symmetry of the current-voltage curve can be assessed.

**Figure 3:**
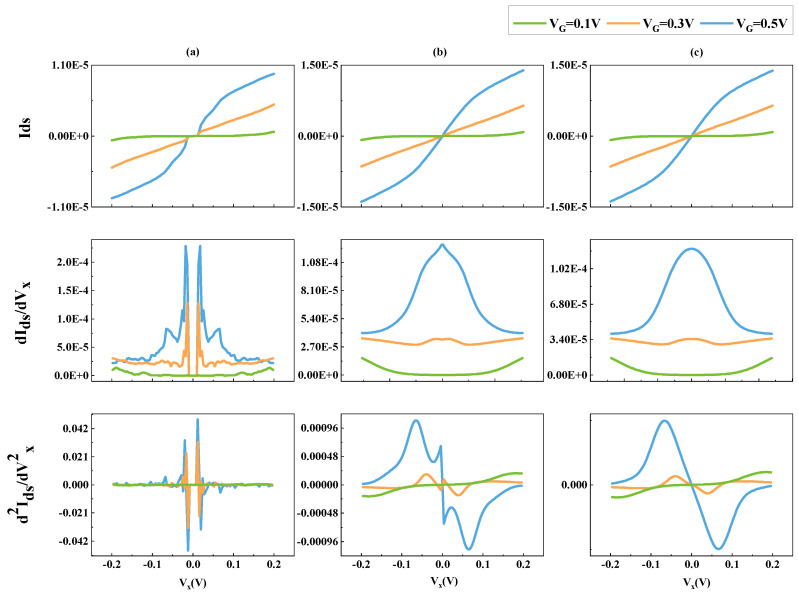
$I_{ds}$, first, and second derivative with aspect to $V_{X}$. (**a**) NN without the smooth loss function. (**b**) NN with the smooth loss function. (**c**) NN with smooth function and domain transform function.

In the field of artificial neural networks, the use of the smooth loss function is beneficial in encouraging the network to generate smooth and continuous predictions as the input values vary. This is particularly important when the output is a function of multiple inputs, as small changes in one input can lead to significant changes in the output. In Figure 3, a comparison is made between two neural networks. Figure 3a shows the NN without a smooth loss function. Figure 3b shows the NN with a smooth loss function. It is observed that the network with the smooth loss function can produce a smooth and relevant current-voltage curve, even for first-order derivatives with respect to $V_{X}$.

However, a stripe is observed in the first-order and second-order derivatives of the predicted curve when $V_{X}$ is near zero. This issue is addressed in the next section using a technique called Feature Domain Transform.

### 2.2. Domain Transform

The paper highlights that the most fundamental physical behavior of a device is zero current. The drain current ($I_{d}$) should be zero when the drain-source current ($V_{DS}$) is equal to zero. Additionally, the $I_{d}$ in both the sub-threshold and saturation regions should achieve high accuracy. However, the sub-threshold $I_{d}$ is too small to distinguish at the normal scale. Klemme et al. [9] used two separate nets, which may introduce discontinuities and non-smoothness near the connection. To address this issue, the paper proposes transforming $I_{d}$ to the logarithmic scale. To ensure physical accuracy, the output of the NN (*y*) is defined in a way that constrains $I_{d}$ to be zero when $V_{DS}$ is zero, irrespective of the NN output. The transforming function is as follows in Equation (3).

(3) $$y=ln(\frac{I_{DS}}{V_{DS}})$$

**Figure 4:**
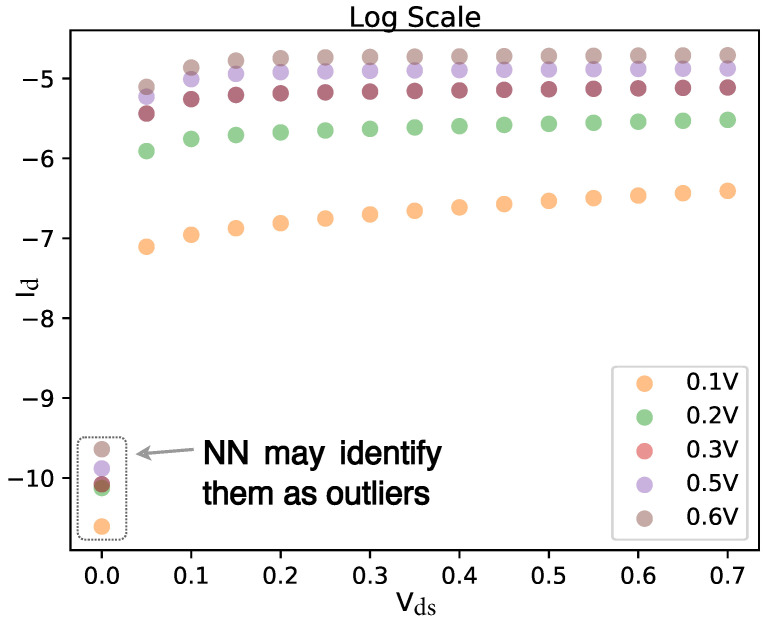
Drain current $I_{d}$ in the logarithmic scale. The points in the left bottom may be identified as outliers.

As mentioned earlier, the output of the NN is in the logarithmic scale, which is essential for sub-threshold region modeling. However, the training data are discretely sampled from TCAD simulation, and the change in $I_{d}$ becomes very sharp in the logarithmic scale when $V_{D}$ approaches zero, as shown in Figure 4. This poses a challenge for the NN as it tends to treat sharp changes as outliers.

To solve this problem, the paper proposes the Domain Transform function for $V_{ds}$ and $V_{gs}$, as shown in Equations (4) and (5), respectively.

(4) $$\begin{array}{cc} V_{ds\_new} & =\mathrm{sign}\left(V_{ds}\right)\times \left({\left(V_{ds}^{2}+\gamma^{2}\right)}^{1/2}-\gamma \right)\\ \gamma & =Q_{1}\left(Range\left(V_{ds}\right)\right) \end{array}$$

(5) $$V_{gs\_new}=V_{gs}+\left(V_{ds\_new}-V_{ds}\right)/2$$

Here, γ represents the first quartile ($Q_{1}$) of the $V_{ds}$ range. This function squeezes $V_{ds}$ in $Q_{1}$ to increase data sample density, which is beneficial for fitting the trend when $V_{ds}$ approaches zero. The $sign(V_{ds})$ factor allows the function to handle both the symmetry of $I_{d}$ with respect to $V_{ds}$ and the unsymmetrical cases [11]. Moreover, to balance the effects of $V_{ds}$ transformation, a bias is added to $V_{gs}$. As the GST results shown in Figure 3c, the $I_{d}$ when $V_{ds}$ near zero is pretty smooth, even for the second-order derivative.

In device modeling, there are several electrical targets that must be achieved. In this paper, we evaluate the error for four targets: threshold voltage ($V_{th}$), saturation drain current ($I_{dsat}$), off-state current ($I_{off}$), and drain current ($I_{ds}$). The metrics used to measure the error are Mean Absolute Error (*MAE*), Mean Absolute Percentage Error (*MAPE*), and Root Mean Square Percent Error (*RMSPE*), as shown in Equation (6).

(6) $$RMSPE\left(I_{ds}\right)=\sqrt{\frac{1}{m}\sum_{i=1}^{m}{\left(\frac{log\left(I_{ds,True,i}\right)-log\left(I_{ds,Pred,i}\right)}{log(I_{ds,True,i})}\right)}^{2}}$$

where *m* is the total number of samples, *i* is the *i*-th sample, $I_{ds,True,i}$ is true $I_{ds}$ value of the *i*-th test point and $I_{ds,Pred,i}$ is the predicted $I_{ds}$ value of the *i*-th test point.

The impact of the γ parameter in Equation (4) on accuracy and complexity is presented in Table 1. As the quartile increases, the errors of $V_{th}$ and $I_{dsat}$ increase. However, if the quartile is less than the second quartile, the error increase in all metrics is limited. The training time remains almost the same, taking into account the influence of running conditions. The prediction time may slightly increase due to the transformation function used.

### 2.3. Monotonic Network Block

The NN-based model lacks the ability to enforce the desired monotonic dependence between input and output, which is commonly observed in device characteristics. For instance, in a device, the drain current ($I_{d}$) exhibits a monotonic relationship with the gate voltage ($V_{g}$), and the on-state current ($I_{on}$) decreases as the gate length ($L_{g}$) increases. To overcome this limitation, a Monotonic Block has been proposed to incorporate the knowledge of monotonic device characteristics into NNs. The weights of the Monotonic Block are non-negative, while the weights of the Normal Block range from negative infinity to positive infinity. The non-negative constraint is enforced by squaring the weights, as illustrated in Equation (7).

(7) $$o_{j}^{l}=\sigma \left(\sum_{k}{\left(W_{jk}^{l}\right)}^{2}o_{k}^{l-1}+b^{l}\right)$$

where $o_{j}^{l}$ is the the *j*-th output of the layer *l*, σ is the active function, $W_{jk}^{l}$ is the *j*-th weight of the layer *l* connected to *k*-th weight of the previous layer, and the $b^{l}$ is the bias of the layer *l*.

**Figure 5:**
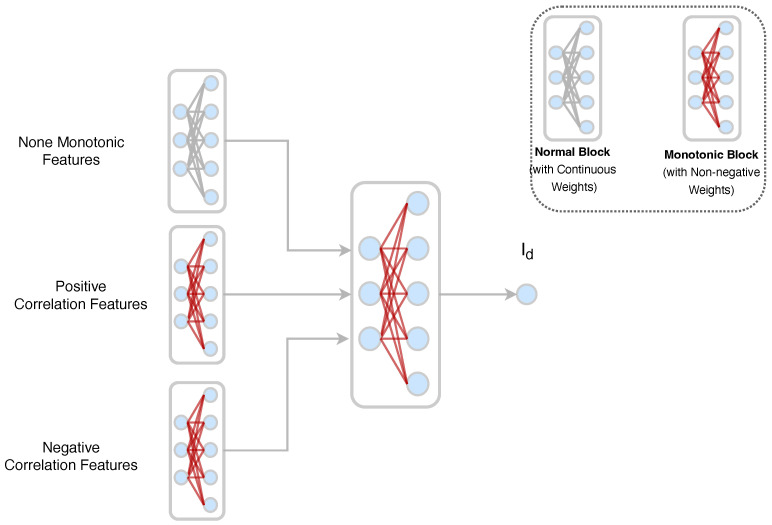
The NN with monotonic block. The block with a gray line represents a normal NN block and the block with a red line represents the monotonic block. The lines in red represent the non-negative weights. The lines in gray represent the continuous values.

The overall NN architecture includes three input groups: None-Monotonic, Positive, and Negative correlation features, which are combined by the Monotonic Block to produce the output $I_{d}$, as shown in Figure 5. This architecture can constrain the correlation effectively to ensure that physical behaviors are not broken, which is critical in device modeling.

### 2.4. Knowledge Transfer

**Figure 6:**
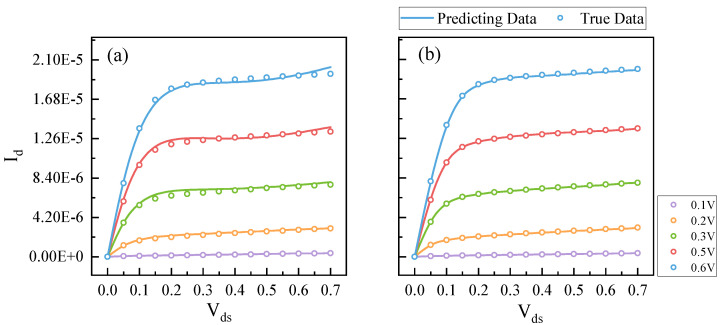
Prediction results of NN. (**a**) Results without knowledge transfer. (**b**) Results with knowledge transfer. The lines represent the prediction results. The dots represent the simulation data.

When a new device is designed, the NN-based compact model cannot leverage previous learning and must train from scratch. This can be time-consuming and the training accuracy cannot be guaranteed. To solve this problem, transfer learning is processed here. By transferring knowledge learned from previous device models to new ones, the training process can be expedited, and the accuracy of the model can be improved. For instance, the knowledge learned from modeling a Planar can be transferred to modeling a GAAFET. Setting the initial weight accordingly can still improve the accuracy of the new model, regardless of whether the NN architecture is the same as the previous one or if only a portion of the architecture is shared. This improvement can be attributed to the shared similarities in their physical behaviors. In Figure 6, transfer learning can significantly improve fitting accuracy after 100 training epochs. To further improve the knowledge transfer quality, some optimization techniques can be utilized, such as fine-turning a portion of layers and a new learning rate scheduling method [20].

## 3. Automatic Neural Network Generation Framework

**Figure 7:**
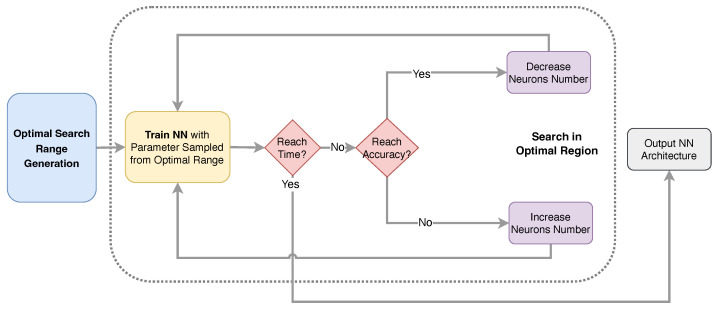
The automatic NN architecture generation flow.

The physical behavior of device modeling is guaranteed by the Physics-Informed Neural Networks (PINN) proposed in Section 2. Another difficulty in NN-based device modeling is achieving accuracy. Obtaining high accuracy often requires considerable time and expertise in Machine Learning (ML), because the different NN parameters have significant impacts on accuracy. To simplify the trial-and-error process, we propose a two-step automatic NN generation flow to obtain an optimal architecture for PINN, as shown in Figure 7. The *Optimal Search Range Generation* first gives a range of NN architecture that is suitable for accuracy. Then, a search in the range with feedback will be performed to find an optimal architecture.

### 3.1. Optimal Search Range Generation

To ensure high accuracy in device modeling, the complexity of the NN architecture must match the complexity of the problem. If the NN is too powerful, it may overfit, while if it is too simple, it may underfit. To reduce the search range and find an appropriate NN architecture, we propose the *Optimal Search Range Generation* in Equation (8), inspired by the Vapnik–Chervonenkis dimension, a neural network learnability metric [21].

(8) $$\begin{array}{cc} N & =\prod_{i=1}^{m}\log_{b}\left(n_{i}+b\right)\times e^{\sqrt{corr_{i}}}/s\\ Range_{l1} & =N\pm max(N\times 0.2,N_{min}) \end{array}$$

Here, *N* represents the center of search range result. *m* is the input feature number, $n_{i}$ is the number of samples for feature *i*, and $corr_{i}$ is the correlation coefficient of the feature *i*. The base factor is denoted by *b* and the scale factor by *s*, and we set 4 as the default. The resulting range of layer one is denoted by $Range_{l1}$, while the range of layer two is half of $Range_{l1}$. The $N_{min}$ is set to 8.

### 3.2. Search in Optimal Region

After determining the optimal search range for the NN architecture, the next step is to find the optimal accuracy while taking into account constraints on prediction time and other criteria. In this search process, meeting the desired prediction time is the primary condition. If the NN achieves the desired accuracy within the given prediction time constraint, the number of neurons is decreased to further reduce the prediction time. On the other hand, if the desired accuracy is not achieved, the number of neurons is randomly increased within the optimal search range in order to improve accuracy.

**Figure 8:**
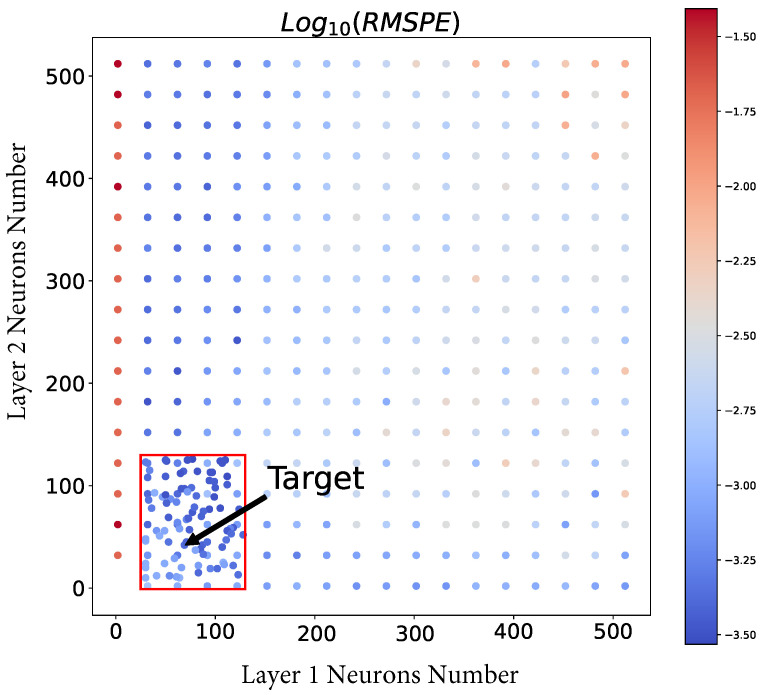
The error changes at the automatic NN structure searching process. The rectangle in red is generated by the *Optimal Search Range Generation* operation. The target is found by *Search in Optimal Region* operation.

The error changes at the AutoNN procedure for GAAFET device modeling is shown in Figure 8, where the red rectangle represents the optimal search range generated using the proposed Equation (8). The final goal is to obtain an optimal architecture that balances accuracy and lightweight. The results demonstrate that the *Optimal Search Range* can provide a suitable NN architecture. Furthermore, it has been observed that having a low number of neurons in the first layer of the NN hampers its ability to extract sufficient information for achieving high accuracy. On the other hand, increasing the number of neurons in the first layer can lead to overfitting, particularly when the NN’s representational power becomes excessively high. Moreover, based on the distribution of loss, it is evident that layer 1 of the NN has a greater influence compared to layer 2. This suggests that the initial layer plays a crucial role in capturing and representing the essential features and patterns in the data, while the subsequent layers may further refine and process this information.

## 4. Experimental Results and Discussion

### 4.1. Environment Setup

Our framework was evaluated on the open-source GAAFET dataset [22]. The values for $V_{dd}$ and $V_{dlin}$ were 0.5 V and 0.1 V, respectively. Table 2 presents the boundaries and sample number for each of the five input parameters. The dataset contained 98,175 samples, which were split into 68,595 samples (70%) for training and 29,580 (30%) for testing.

### 4.2. AutoPINN Physical Behaviors

To check the smoothness, Figure 3 exhibits the Gummel Symmetry Test results of different NNs. The results of the default NN implemented in Pytorch targeting tabular data [23] are shown in Figure 3a. This is a NN-based model without physical information embedded. It is suffering unphysical behaviors: $I_{d}$ is not smooth and differentiable, and it is not monotonic, meaning it does not consistently increase or decrease, and it reaches zero at an early stage when $V_{ds}$ is not zero. After some physical information is embedded, the prior work [10] shows better results. However, it is also unsmooth near $V_{ds}$ = 0, as shown in Figure 3b. Ours shows promising physical behaviors as shown in Figure 3c.

**Figure 9:**
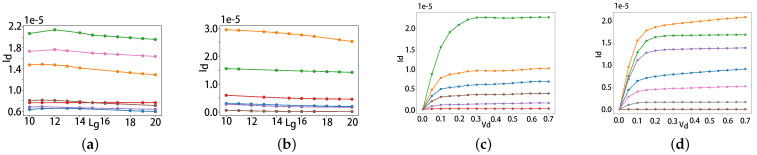
Relationship between (**a**) $I_{ds}$ and $L_{g}$ without monotonic block. (**b**) $I_{ds}$ and $L_{g}$ with monotonic block. (**c**) $I_{ds}$ and $V_{ds}$ without monotonic block. (**d**) $I_{ds}$ and $V_{ds}$ with monotonic block. The index is a randomly selected data sample number in the public dataset [22].

To check the monotonic, Figure 9 shows the monotonic relationship between inputs and output. After adding a negative constraint on $I_{ds}$ and $L_{g}$, the curve becomes monotonic and smooth from Figure 9a,b. Figure 9c,d show the relationship between $V_{ds}$ and $I_{ds}$ without and with monotonic block. The block obviously solves the no-monotonic at saturation region.

### 4.3. AutoPINN Accuracy

**Figure 10:**
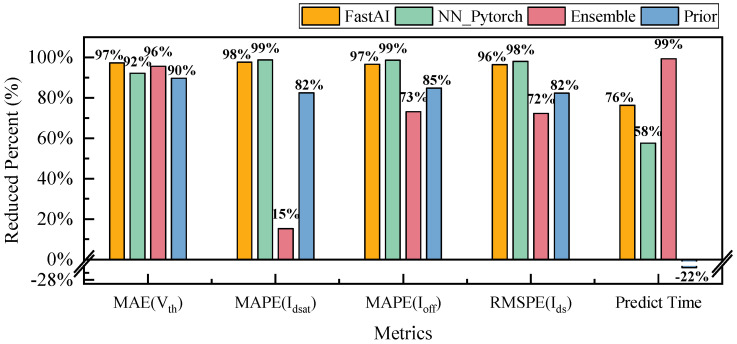
The reduced percent compared to FastAI, NN proposed by Pytorch, and Ensemble learning.

Our framework was compared with several existing models that specifically target universal tabular data. These models include FastAI [24], Pytorch NN model [23], and the ensemble learning model released by Autogluon [25]. Additionally, we compared our framework with the prior work released by Tung et al. in 2022 [10]. Table 3 shows the experimental results. Compared to the best-performing model among them, our AutoPINN can reduce the MAE of $V_{th}$ by 95%, and MAPE of $I_{dsat}$ and $I_{off}$ by 15% and 73%, respectively. In addition, the total curve of $I_{ds}$ can be reduced by 72%, as shown in Figure 10.

The total $I_{ds}$ curve error can be substantially reduced to 0.05% by our AutoPINN. It should be noted that other machine learning algorithms have a tendency to perform un-physical behaviors that are not acceptable for real applications. The prior work [10] was evaluated using their default settings. The accuracy of it is less than the ensemble learning. It reflects that the AutoNN is necessary to obtain high accuracy.

Prediction time is also significantly improved. When evaluated on the 29,580 test data samples with 116 calls, each call evaluating 255 samples, our AutoPINN only took 0.85 s to execute. Ours reduced the time by 99% compared to the ensemble learning model. The multiple calls used here aim to account for the warm-up time of the model.

### 4.4. AutoPINN Generalization

The generalization refers to the ability to fit unseen data, i.e., test data. The generalization problem arose from the training and testing data usually having different distributions. There are two methods to evaluate the generalization of the NN. One is the NN accuracy on test data, which is the most important metric. Another is the loss landscape. The NN accuracy on test data is promising in Section 4.3. This section mainly talks about the loss landscape to reveal the generalization of NN.

The loss landscape is an intuitive way to visualize the generalization. The NN with a flat loss landscape has a better generalization than the sharp one [26,27]. To generate the loss landscape, Equation (9) is a widely adopted method.

(9) $$\tau \left(\alpha ,\beta ;\theta^{*}\right)=L\left(\theta^{*}+\alpha \delta +\beta \eta \right)$$

where $\theta^{*}$ is the normalized weight of trained NN, and δ and η are two random directions of the parameter $\theta^{*}$. The α and β are the factors applied in two directions. They control how far away from original parameter $\theta^{*}$. Varying α and β from −1 to 1 is used to obtain the loss and draw the loss landscape.

**Figure 11:**
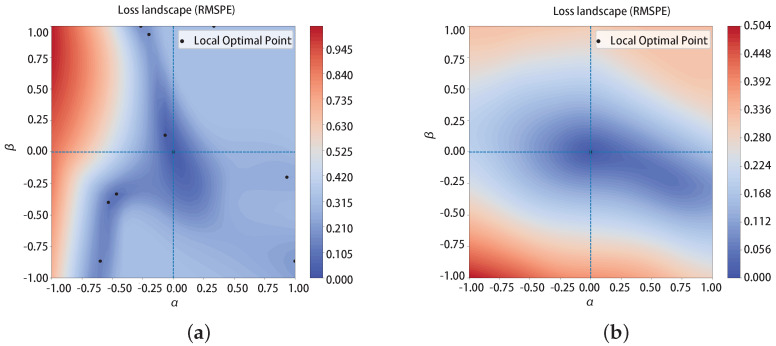
Loss landscape visualization. The black dots represent local optimal points. (**a**) Loss landscape of a not well-optimized Neural Network structure; (**b**) loss landscape of our well-optimized Neural Network structure.

Figure 11a presents the loss landscape of a not well-optimized Neural Network structure, i.e., (5,8,8,8,1). The minimum number of this loss landscape is ten while our well-optimized NN only has one minimum, as shown in Figure 11b. The more local minimum number in the loss landscape, the harder to achieve convergence in the training process. Ours is also flatter than the not well-optimized NN. The good accuracy on test data and the flat loss landscape shows that our NN model can achieve high generalization.

## 5. Conclusions

This paper presents a novel framework called AutoPINN for NN-based semiconductor device modeling. AutoPINN solves two major challenges: unphysical behaviors and the requirement for NN expertise. The framework consists of two components: PINN and AutoNN.

PINN is introduced to tackle unphysical issues by incorporating physical information using several key technologies. There are a few key technologies used here. The *Domain Transform* ensures that the current–voltage relationship ($I_{d}$ vs. $V_{gs}$ and $V_{ds}$) is smooth and differentiable by transforming them with higher density near $V_{ds}=0$. This transformation handles both symmetric and asymmetric drain/source scenarios. It also transforms the optimization target to simplify NN fitting. The *Smooth Loss Function* considers not only the optimization target, but also derivatives and other factors to ensure a smooth and differentiable $I_{d}$ curve. The *Monotonic Network Block* enforces non-negativity constraints on the NN weights to achieve monotonic behavior. *Knowledge Transfer* enables the transfer of modeling information and training from other devices, facilitating faster training convergence and improved physical behavior.

AutoNN assists PINN in finding an optimal structure without requiring human expertise. It generates an optimal search range for the NN architecture and optimizes accuracy while considering constraints such as prediction time and other criteria.

The effectiveness of the AutoPINN framework is demonstrated through experiments on a GAAFET device. The results show high accuracy while maintaining a lightweight model. To ensure generalization, validation results on sample data as well as the loss landscape are utilized to confirm the approach’s ability to generalize well. The authors believe that this work has the potential to accelerate the development and simulation processes of emerging devices.

## Figures and Tables

**Table 1:** Experimental results on different γ parameter.

Metrics	No Transform	First Quartile (Ours)	Second Quartile	Third Quartile
$MAE\;(V_{th})$	0.6	0.9	2.3	2.7
$MAPE\;(I_{dsat})$	0.37%	0.35%	0.65%	0.62%
*$MAPE\;(I_{off}$)*	1.30%	1.22%	1.30%	1.28%
$RMSPE\;(I_{ds})$	0.05%	0.05%	0.06%	0.06
Training Time (s)	234.05	217.88	223.6	231.4
Prediction Time (s)	0.71	0.85	0.8	0.83

**Table 2:** The electrical parameters boundaries and sample number of a GAAFET dataset.

Parameters	Lower Boundary	Upper Boundary	Sample Number
$lg_{nw}$	10	20	11
$r_{nw}$	2	5	7
$t_{ox}$	0.5	1.5	5
$V_{g}$	0	0.7	15
$V_{d}$	0	0.7	15

**Table 3:** Experimental results compared to FastAI, NN proposed by Pytorch, and Ensemble learning.

Metrics	FastAI [24]	NN_Pytorch [23]	Ensemble [25]	Prior [10]	Ours	Reduced Percent Compared to Ensemble
** $MAE\;(V_{th})$ **	0.0351	0.0118	0.0209	0.009	0.0009	95.6%
** $MAPE\;(I_{dsat})$ **	0.1487	0.2865	0.0041	0.02	0.0035	15.2%
** $MAPE\;(I_{off})$ **	0.3542	0.9003	0.0454	0.08	0.0122	73.1%
** $RMSPE\;(I_{ds})$ **	1.48%	2.69%	0.19%	0.3%	0.05%	72.2%
Training Time (s)	38.23	301.30	476.10	124	217.88	54.2%
Prediction Time (s)	3.61	2.01	123.25	0.7	0.85	99.3%

## Data Availability

The training and testing dataset is available on GitHub [22]. The other data presented in this study are available on request from the corresponding author.

## Abbreviations

The following abbreviations are used in this manuscript:

ML	Machine Learning
ANN	Artificial Neural Network
NN	Neural Network
AutoNN	Automatic Neural Network Generation
PINN	Physical-Informed Neural Network
AutoPINN	Automatic Physics-Informed Neural Network Generation
$L_{g}$	Length of Gate
$r_{nw}$	radius of nanwire
$t_{ox}$	Thickness of oxide
$V_{g}$	Gate Voltage
$V_{d}$	Drain Voltage
